# Promoting adherence to treatment for latent TB infection through mobile phone text messaging: study protocol for a pilot randomized controlled trial

**DOI:** 10.1186/s40814-017-0128-9

**Published:** 2017-03-13

**Authors:** Eyal Oren, Melanie L. Bell, Francisco Garcia, Carlos Perez-Velez, Lynn B. Gerald

**Affiliations:** 1 Department of Epidemiology and Biostatistics, College of Public Health, University of Arizona, 1295 N. Martin Avenue, P.O. Box 245211, Tucson, AZ 85724 USA; 2 Department of Health Promotion Sciences, College of Public Health, University of Arizona, 1295 N. Martin Avenue, P.O. Box 245211, Tucson, AZ 85724 USA; 3 Pima County Health Department, University of Arizona Health Sciences, Tucson, AZ USA; 4 Asthma and Airways Disease Research Center, University of Arizona Health Sciences, Tucson, AZ USA

**Keywords:** Latent tuberculosis infection, Text messages, Treatment adherence

## Abstract

**Background:**

An estimated two billion people, over one third of the world’s population, have latent infection with *Mycobacterium tuberculosis* (LTBI). Patient adherence to LTBI treatment is currently poor given that individuals show no symptoms of illness and may not feel that they are at risk of developing active tuberculosis (TB). Short text messages can serve as a simple reminder to take medications and address barriers to adherence such as forgetfulness and lack of social support.

**Methods/design:**

We aim to determine the feasibility and acceptability of text reminders for improving adherence in latent TB patients using a randomized controlled single-blinded trial, measuring adherence through an increase in treatment completion rates. Forty adult LTBI participants will be randomized to either text messages plus phone call reminders or phone call reminders only (usual care). Recruitment, retention, and study acceptability will be assessed as primary outcomes.

**Discussion:**

This pilot study will examine the feasibility of using text messaging for increasing adherence to treatment for latent tuberculosis infection. The study will allow for evaluation of process measures and challenges and development of a model for scaling up an effectiveness trial for increasing treatment adherence.

**Trial registration:**

NCT02690818 (Clinical Trials.gov)

**Electronic supplementary material:**

The online version of this article (doi:10.1186/s40814-017-0128-9) contains supplementary material, which is available to authorized users.

## Background

Tuberculosis (TB) is a leading cause of death from an infectious disease worldwide. An estimated two billion people, one third of the world’s population, are infected with *Mycobacterium tuberculosis*, resulting in approximately 1.4 million deaths annually [1]. People with untreated or undiagnosed TB who do not complete their prescribed treatment regimen pose major health risks to themselves and to the general population. Those patients who do not adhere to treatment for active TB remain infectious longer and are more likely to relapse and die. They are also vulnerable to developing drug-resistant tuberculosis [2].

### Adherence to latent tuberculosis infection therapy

Approximately one third of individuals exposed to someone with active TB disease develop latent TB infection (LTBI) [3]. Patients who develop LTBI are asymptomatic and non-contagious but the overall lifetime risk of LTBI progression to active TB is estimated at approximately 5–10% [4]. This risk is greatly increased among immunosuppressed individuals, including those with HIV, diabetes, and heavy steroid use [5, 6]. The populations with the highest burden of LTBI in the USA are primarily from immigrant, refugee, and non-English speaking communities. Ten million individuals in the USA, including 19% of foreign-born residents, are estimated to have LTBI [7, 8]. One approach to reduce the burden of TB is to detect and treat persons while they still have LTBI to prevent disease development. This approach is recommended for individuals at increased risk for progression to disease [9]. These include persons who were born in countries with increased tuberculosis prevalence and persons who live in high-risk congregate settings. Patient adherence to LTBI therapy is low as LTBI is asymptomatic, patients may not understand risk of developing active TB, LTBI therapy has potential side effects, and LTBI therapy is of long duration (usually 9 months) [10–12]. In a large prospective North American study, lower treatment completion rates were noted among females, US-born, Hispanic, and uninsured populations [13]. As a result, the proportion of persons who complete LTBI treatment in the USA ranges between 39 and 65%. These low adherence rates undermine the potential benefits of preventing TB progression in individuals with LTBI and maximizing TB control from a public health standpoint [14, 15]. Low adherence also increases the burden of active TB disease within the community and can increase the rate of drug-resistant TB strains.

One of the major shortcomings in TB control thus remains the difficulty in reaching marginalized populations for the purpose of TB prevention [16]. The ability to follow treatment plans in an optimal manner is frequently compromised by multiple barriers. Others have noted some of these barriers, including younger age, lower perceived risk for TB disease, and self-administered regimens [17]. Measures to improve adherence aim to (1) help patients address social issues which may hinder their ability to follow treatment, (2) enhance contact with health professionals, and (3) strengthen communication between health professionals and patients during treatment [18]. The best way to improve adherence to long-term treatment regimens may be through extended supervision of patients [19]. However, direct supervision of treatment is expensive and has mixed results in increasing LTBI treatment completion [20, 21]. Reminder trials for TB have demonstrated benefits on adherence to appointments [22]. Yet, treatment adherence and completion rates remain suboptimal across high-risk groups, and no intervention has shown consistent effectiveness [15]. For example, the role of education as a predictor of adherence has not been well-defined [15]. Similarly, the effect of immediate compared to deferred monetary incentives on adherence has not been shown to significantly differ [23].

### Potential benefits of text messaging

Over two trillion text messages were sent in the USA in 2011, with more than 80% of the US population owning a mobile phone and almost 70% of these phone owners regularly sending or receiving text messages [24]. Unlike the digital divide with online technologies, people of color and low-income individuals are more likely to text [25]. With this widespread adoption, texting offers promise for public health departments to make health information more readily accessible. Texting allows quick, direct communication that works on all types of mobile phones. Texting can be used in conjunction with other initiatives to establish and maintain contact between the patient and provider over the course of treatment. The information-motivation-behavioral skills model demonstrates that information is a prerequisite for good adherence, yet it is not sufficient in itself in explaining behavior [26]. Mobile phones can benefit both patients and providers by overcoming resource limitations, structural barriers, and behavioral limitations in providing motivation and the development of desired behavioral responses [27]. Conceptually, text messaging relies on constructs from the Social Cognitive Theory where positive outcome expectations, e.g., beliefs about the likelihood and value of the consequences of taking medication, as well as self-efficacy (beliefs about personal ability), substantially contribute to forming an intention to perform a desired action [28]. For example, participants in a multisite randomized clinical trial of HIV-infected adults initiating antiretroviral therapy asked to respond to a text message indicating that they had taken their medications were more likely to take their medications [29, 30]. Text messages have the advantage of being efficient and are considerably less invasive to daily lives compared to phone calls [31]. Mobility, instantaneous access, and direct communication are likely to enhance efficiency of service delivery [32, 33]. With increased efficiency, cost savings are substantial and include direct costs defrayed to the health system as well as opportunity costs of missed appointments and lengthy treatment duration [34, 35].

A stronger evidence base needs to be developed around the mobile health field for TB, before effective approaches can be implemented and high-quality data are lacking [36, 37]. Only recently have studies investigated the effectiveness of texting reminders for active tuberculosis treatment [34, 38, 39]. To our knowledge, no studies have been completed and only one study is currently being carried out in the context of latent TB infection [40]. As such, tailoring a reminder system for LTBI patients who self-administer medications could improve adherence to LTBI treatment and provide evidence for adopting or expanding reminder systems. A commonly used practice for LTBI is a phone call to remind patients to visit the clinic for a check-in and monthly refill. Since we would like to assess the independent effect of text messages on treatment adherence, we propose to evaluate the use of daily text messaging, in addition to the reminder phone call. We propose a study examining the feasibility of a randomized controlled trial using text messages to improve LTBI treatment adherence. This study will provide us the necessary data to design a large randomized controlled trial which will examine the effectiveness of text messaging.

### Study objectives

We aim to determine the feasibility and acceptability of text reminders for improving adherence in latent TB patients. Specific objectives of the current study are to (1) assess the feasibility of the intervention as indicated by participant recruitment and retention and (2) evaluate the acceptability of the intervention, as indicated by intervention adherence, outcome measurement rates, and feedback from participants.

Data will be used to design a definitive trial which will test the hypothesis that the intervention will improve medication adherence, as measured through an increase in treatment completion rates, and result in higher self-reported medication adherence, fewer missed appointments and doses, and a shorter course of treatment.

## Methods/design

### Overview

This is a single-blinded randomized controlled parallel trial to test the feasibility of using text messages to improve LTBI treatment adherence. Randomization will be performed with a 1:1 allocation. The project is funded through the American Lung Association and is being led by researchers at the University of Arizona with recruitment and collaboration taking place at the Pima County Health Department (PCHD) TB Control Program in Tucson, AZ, USA. This manuscript adheres to the SPIRIT checklist reporting guidelines (Additional file 1).

### Participants

Forty participants from PCHD are being recruited, with adequate representation expected from samples previously noted to fail treatment [13]. Pima County Health Department prioritizes LTBI screening based on recent contact to an infectious TB patient, migration from a high-burden TB country, as well as the USPSTF guidelines [9]. Persons who are at least 18 years of age and able to provide informed consent, agree to initiate LTBI treatment, are prescribed self-administered 4-month rifampin or 9-month isoniazid therapy, and have a phone that can receive text messages, will be eligible for the study. Participants will also need to be able to communicate via text messaging or have a family member or friend able to provide assistance with messages for the duration of the study. Patients are ineligible if they currently have active tuberculosis disease. Participants from the pilot will not be included in a proposed larger follow-up study.

### Participant recruitment and consent

All participants will be recruited directly from the PCHD Tuberculosis Control Program. A clinic nurse will refer interested participants to study personnel, at which point these personnel will assess eligibility and obtain informed consent. The referral will occur when a patient agrees to begin treatment for LTBI. Participants will be informed through the consent process that they may withdraw from the study at any time for any reason without it affecting their medical care. Participants will also be provided the purpose of the study, study objectives, as well as how study success will be measured [41]. The informed consent document will use simple language to minimize barriers posed by low literacy. A language line will also be used to verbally explain study materials, assist with language difficulties, and overcome reading difficulties. Family members or friends who are present at the time of recruitment will also be utilized for support and translation, but will not take the place of the language line.

Eligible individuals will be recruited over a 12-month period. Following consent, all participants will complete baseline assessments and will be randomly allocated to group.

### Randomization and allocation

Eligible patients who have given informed consent and completed baseline assessments will be randomly assigned to receive either text messaging and usual care or usual care only. A list allocating participants to the intervention or control arm will be generated by the biostatistician (MB), who has no patient contact, using the statistical software R. Randomized blocks of variable size will be used to ensure balance between the arms. Block size will be random to reduce predictability of the sequence. Each individual will be assigned a unique study code, which will be used throughout the research study in place of any identifying information. Participant identification and allocation will be placed in sealed opaque envelopes. Allocation codes will be concealed until study group is assigned. Participants will be identified to the research team only by phone number and study code.

### Blinding

Investigators will be masked to group allocation, but participants and research staff will not, given their active study roles.

### Usual care

Standard of care for LTBI patients at this clinic includes an initial discussion of medication risks, benefits, and side effects. Treatment usually involves daily self-administration of medication for a prolonged period of therapy. Treatment typically involves taking rifampin 600 mg daily for 4 months or isoniazid 300 mg daily for 9 months. If a patient agrees to treatment, they are given a 30-day supply of medication. Subsequent follow-up involves monthly clinic visits, at which time there is an assessment of symptoms, side effects, and adherence; blood-work is performed; and another month’s supply of medication is provided. The usual practice of care with regard to adherence is a phone reminder prior to the next appointment.

### Intervention

Text group participants will receive a daily text reminder sent by an automatic messaging system in addition to the standard of care. This automation will anonymize messaging to all but the study coordinator, who sends out the message. Participants will be asked optimal times to receive daily texts and will receive texts in their language of choice. Participants’ phone numbers will be entered into a secured web platform to automatically send text message reminders at pre-specified intervals. A delivery report function will be used to verify that messages were received and opened. The system meets the key requirements of a highly functional texting system, including customization, personalization, message notification, and remote access [42]. The script sent to individuals will state “This is a reminder to take your medication. Please respond.” The Platform for Research Integrated Messaging (PRiM), a web-based software suite developed at the University of Arizona, allows text messaging integration and management of text messages. It is hosted on secure servers, includes only de-identified data and delivered as SaaS (Software as a Service). To the user, text messages appear to come from a specific and established phone number. Participants will use existing mobile phone services; while phones and network airtime will not be paid for, participants will be provided a $40 incentive, paid at the 2-month visit and at final survey completion. Participants can request to opt out of the intervention at any time by calling or texting “stop.”

### Outcome measures

All outcomes will be assessed through in-person participant visits or collection from clinic records. Questionnaires will be completed by trained interviewers who are blinded to participant allocation.

The primary feasibility outcomes are summarized in Table 1.

**Table 1:** Feasibility outcomes

Outcome	How assessed	Target
Recruitment	How many approached by clinic, how many referred, how many study eligible, how many consented; why ineligible or refused; how long to recruit each patient	40% recruitment rate, based on recruitment of all eligible
Retention	How many drop out at each monthly time point, who and why	70% retention rate
Patient satisfaction and acceptability	Patient perceptions, satisfaction, and acceptance of the intervention through pre- and post-surveys	75% satisfaction rate based on positive responses to questionnaires

While this pilot study will not have the power to detect differences in efficacy, efficacy outcomes will be measured to test procedures for early indication of a promising intervention. The primary efficacy outcome is the difference in proportion of participants completing treatment in each of the study groups. Successful treatment completion is defined as taking at least 80% of the doses of rifampin prescribed within 20 weeks [43]. Estimates of treatment completion will be used to indicate whether the treatment appears promising and to estimate sample size for a future definitive trial. Additional secondary outcomes include proportion of missed visits out of total scheduled visits; the number and length of delays in medication refill; number of missed doses; overall length of treatment (time to dropout); and self-reported adherence. While potentially overestimating true adherence, self-reported adherence is a robust measure of adherence [44, 45]. A brief pre-survey and more detailed semi-structured interviews will be conducted with all individuals to qualitatively assess preferences, attitudes, and satisfaction with the study, as well as barriers to adherence.

**Fig. 1:**
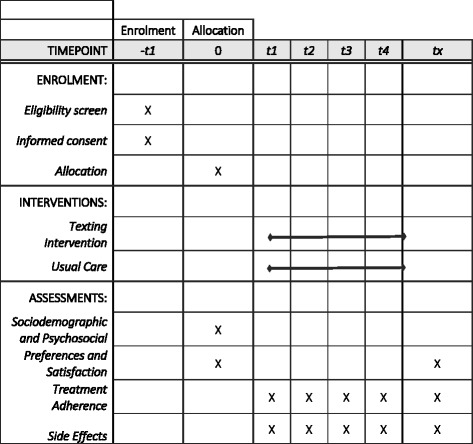
Schedule of enrolment, interventions, and assessments

The following assessments will be completed by participants (Fig. 1), as per the SPIRIT Statement [46].

### Sample size

The sample size of 40 will yield a 5% margin of error (95% confidence interval half-width) of no greater than 7.7%, for any of the binary feasibility outcomes (recruitment, retention), using the formula $p\pm 1.96\sqrt{\frac{p\left(1- p\right)}{n}}$ for a single binary proportion and the value of *p* which maximizes the standard error (*p* = 0.5). A sample size of *n* = 40 participants [47] is expected to provide data sufficient to estimate variability in outcome measures as well as to assess trial feasibility. Furthermore, Whitehead et al. have shown that 40 participants in a pilot study are optimal when the future main trial is expecting a small to medium effect size [48]. We also expect qualitative data to be saturated with 30 interviews, at which we expect to yield sufficient data to achieve redundancy. However, we will increase the sample size until saturation is reached if needed [49].

### Data collection, management, and analysis

**Fig. 2:**
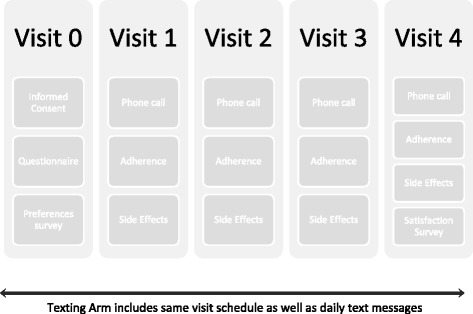
Sample visit protocol, 4-month regimen

Trained interviewers will collect socio-demographic information and clinical data through in-person participant visits or collection from clinic records for all participants, including adherence rates (dose counts, appointments) at baseline and then on a monthly basis. Additionally, information will be collected at baseline regarding sharing of phones, baseline perceptions of texting, and frequency of texting. Per usual care, participants are expected to return to clinic once a month for a check-in and medication refill. Self-reported missed doses and pill bottle counts are recorded in the medical chart and will be abstracted to a study monitoring log. Figure 1 shows the various data elements collected at study time points and Fig. 2 a sample visit protocol for the 4-month regimen.

Participants will be prospectively followed until treatment completion or dropout. We will define participants as lost to follow-up if they are unable to be traced per clinic protocol (two phone calls and a certified letter). The research coordinator will maintain a log documenting refusals and reporting recruitment numbers.

Detailed semi-structured interviews will be conducted with all individuals during their final visit to qualitatively assess preferences, attitudes, and satisfaction with the study intervention. User attitudes will be assessed for both the texting intervention and phone reminder system. Text group participants will be asked for reasons for non-response. These might include forgetting to text back, being too busy, personal reasons, or not understanding the protocol. Treatment defaulters, as assessed through chart review, will be asked for non-adherence reasons and satisfaction with various reminder systems through a one-time phone interview. All interview guides will have been piloted during staff training and presented by an interviewer familiar with the language or else with the aid of an interpreter service. Participants will be asked whether messages are useful and acceptable, whether they found the message text and format to work, and are of preferred quantity, frequency, and timing of use. Information will also be collected to better understand initial expectations and whether attitudes had changed towards the intervention throughout the process. In addition, they will be asked whether they would like to keep using the text message reminders after the study and whether they would recommend the text message reminder system to others. We anticipate including some redundancy of questions to assess internal consistency of responses. We will also collect (a) demographic characteristics of participants, (b) perceived benefits of, and barriers to, LTBI treatment completion, (c) self-efficacy to complete treatment, (d) perceived susceptibility to TB disease, (e) satisfaction with provider interactions and patient education, and (f) preferred reminder system, including whether phone sharing might have affected the intervention and perceptions of costs.

Study data will be collected and managed using REDCap (Research Electronic Data Capture) electronic data capture tools hosted at the University of Arizona’s College of Medicine. REDCap is a secure, web-based application designed to support data capture for research studies, providing (1) an intuitive interface for validated data entry; (2) audit trails for tracking data manipulation and export procedures; (3) automated export procedures for seamless data downloads to common statistical packages; and (4) procedures for importing data from external sources. All data will be password-protected and de-identified [50]. Data will be entered at the time of collection, and clinic data will be abstracted by the clinic coordinator and integrated into the database. To facilitate analysis, the database will include the ability to generate custom reports exportable to other applications. Summary reporting of data will be conducted monthly to ensure satisfactory data quality. Regular meetings will be held with co-principal investigators to assess progress of data collection.

The database will also be used to capture lack of response and number of approach attempts. Individual response rates will be calculated as the number of user responses divided by the total number of surveys sent out. All data will be kept on secured servers. Questionnaires will be administered in confidentiality and responses kept in a secured office.

### Statistical methods

Analyses regarding feasibility of the study will primarily be descriptive in terms of recruitment and retention rate, reasons for dropout, and deviations from protocol. Participation bias will be assessed by comparing individuals included in the study to study refusals as well as broader clinic participant characteristics.

Qualitative data will be transcribed and analyzed for patterns and themes using NVivo 10, a qualitative analysis program with which a member of the team has substantial experience [51, 52]. Answers to open-ended questions regarding barriers and benefits of the reminder systems will be categorized and described.

We will compare the proportion of participants completing treatment in each of the study groups in order to assess preliminary efficacy [43]. Results will be reported as the number (%) of participants for each treatment group, the risk difference, and 95% confidence intervals. Secondary outcomes will include proportion of missed visits out of total scheduled visits; the number and length of delays in medication refill; number of missed doses; overall length of treatment (time to dropout); and self-reported adherence.

Rescheduled or canceled appointments will not be considered as missed although rate of canceled or rescheduled appointments will be collected. Comparisons of the intervention group with the control group for all continuous or count outcomes will be carried out using *t* tests. Proportions will be compared using a non-continuity-corrected test of proportions. Time to dropout will be described using survival analysis methods.

### Methods: monitoring

#### Data monitoring and harms

Due to the minimal risks of the study and nature of the intervention, there is no formal Data Safety Monitoring Board. On a monthly basis, the two study arms will be compared for event rates, including side effects and their severity, as well as distinguishing medication-related from other side effects.

#### Auditing

All research staff have received human subjects research training and will receive appropriate training in recruitment, data collection, and management. Ongoing study progress will be tracked with regular monitoring by the PI with study and clinic personnel, training of all staffs involved in the process, monthly research team meetings, and regular review to ensure that study targets are being met.

#### Confidentiality

The web management application (PRiM) is password-protected, and data can be stored in encrypted format when texting other subjects. Texts are addressed to de-identify usernames, and pass through the website, thus keeping identities private—other subject’s phone numbers are never seen by subjects—as all contacts are indirect through the web application. Only HIPAA-trained researchers are allowed to access or download the data via the website.

## Discussion

Treatment adherence and completion rates for latent tuberculosis infection remain suboptimal across high-risk groups. This study is the first, to our knowledge, to assess the feasibility and acceptability of text messaging as an adjunct to usual care for treatment for LTBI in the USA. Strengths of this study include an adequate recruitment period, provision of an incentive that will help balance concerns related to messaging costs, and in-depth and ongoing collection of process measures.

Some limitations and challenges of this study are the messaging cost for participants, the risk of participants running out of plan minutes, and the possibility that participants change numbers or service providers. Voice calling and text messaging are equally affected by changing phone numbers, and text messaging might be advantageous if a patient is running low on plan minutes. We will recommend that the clinic ask participants about their phone plan at each visit. Participants may miss appointments no matter the intervention, and we recognize the possible limitations of self-reported adherence, including the fact that reporting during the week prior to an appointment may not reflect adherence patterns over time, since participants may become more adherent right before an appointment [53]. Finally, there is possible selection bias of enrollees, since participants must have a phone and be willing to text. Participants may also need to be comfortable with the communication style in order to effectively show behavior change [27]. We will compare our demographic and clinical data to those of non-enrollees, in order to ensure generalizability of our findings.

As a result of this pilot study, we will advance knowledge regarding best practices regarding adherence for treatment of latent TB infection. As this is a pilot study, we recommend results be interpreted with caution. Further intervention studies should verify the results of this study and scale up the proposed approach to broader populations and clinical sites.

## Additional files

Additional file 1: SPIRIT checklist reporting guidelines. (DOC 121 kb)

## Abbreviations

LTBI: Latent tuberculosis infection
PCHD: Pima County Health Department
PRiM: Platform for Research Integrated Messaging
REDCap: Research electronic data capture
TB: Tuberculosis
